# Prevalence and Importance of Tricuspid Valve Prolapse in Patients with Primary Mitral Regurgitation

**DOI:** 10.3390/jcdd13030106

**Published:** 2026-02-24

**Authors:** Aniek L. van Wijngaarden, Anton Tomsic, Nadeem Elmasry, Hoi W. Wu, Meindert Palmen, Jeroen J. Bax, Nina Ajmone Marsan

**Affiliations:** 1 Department of Cardiology, Leiden University Medical Center, 2333 ZA Leiden, The Netherlands; a.l.van_wijngaarden@lumc.nl (A.L.v.W.);; 2 Department of Thoracic Surgery, Leiden University Medical Center, 2333 ZA Leiden, The Netherlands

**Keywords:** mitral valve prolapse, tricuspid valve prolapse, primary mitral regurgitation, echocardiography

## Abstract

The presence and impact of tricuspid valve (TV) prolapse in patients with mitral valve (MV) prolapse and severe mitral regurgitation (MR) have not been widely reported. The aim of our study was to describe the prevalence of TV prolapse, and the associated echocardiography features, in a large cohort of patients with MV prolapse undergoing surgery, and to explore its potential clinical impact. A total of 803 patients were included, of which 87 (11%) were diagnosed with TV prolapse, while 716 (89%) patients showed no TV prolapse. Patients with TV prolapse were more often diagnosed with Barlow’s disease compared to patients without TV prolapse, and also had more frequently significant TR, a larger right chamber size and TV annulus; they also underwent concomitant TV annuloplasty more often. During follow-up, there was no difference in terms of TR progression or all-cause mortality after surgery between the patients with or without TV prolapse. In conclusion, TV prolapse was associated with a more severe phenotype in terms of baseline cardiac remodeling and TR severity in our large study cohort of MV prolapse patients undergoing MV repair. However, when successfully treated, TV prolapse was not associated with worse outcomes after surgery, also in terms of TR progression.

## 1. Introduction

Tricuspid valve (TV) prolapse has long been considered a sporadic condition, but more recent studies have made an attempt to describe its prevalence and observed, in particular, a frequent combination with mitral valve (MV) prolapse. This combination may suggest that the myxomatous degeneration affecting the MV can also involve the TV, especially in more severe phenotypes such as Barlow’s disease, which is also characterized by a larger TV annulus and right chambers [1,2,3]. The reported percentage of coexistence of MV and TV prolapse varies from 5% to 55% [1,2,4,5,6,7,8,9]. Therefore, Lorinsky et al. [6] proposed a standardized definition of TV prolapse using transthoracic echocardiography, and this definition was based on the maximal systolic atrial displacement of the TV leaflets from the tricuspid annular plane to the belly of the leaflets. This definition was then also applied to transesophageal echocardiography and magnetic resonance imaging by subsequent studies, including patients with primary mitral regurgitation (MR). Margonato et al. [2] explored the prevalence and different etiologies of tricuspid regurgitation (TR) in patients with severe primary MR and found that 55% of the patients had echocardiographic characteristics of TV prolapse. By using the same proposed criteria with magnetic resonance imaging, Guta et al. [7] observed TV prolapse in 36% of patients with degenerative MR. However, the implications of this structural alteration of the TV in terms of severity and possible progression of TR and clinical outcomes have not been studied thus far in patients with MV prolapse and severe MR.

Therefore, the aims of the current study were the following: (i) to assess the prevalence of TV prolapse and the associated echocardiography features in a large cohort of patients with MV prolapse and significant MR undergoing surgery, and (ii) to explore the potential impact of TV prolapse on TR before and after surgery, and its association with long-term mortality.

## 2. Materials and Methods

### 2.1. Study Cohort and Clinical Characteristics

Patients who underwent MV surgery for significant primary MR due to myxomatous degeneration at the Leiden University Medical Center (Leiden, The Netherlands) between 2000 and 2020 were included. The following exclusion criteria were used: (1) unavailable pre-operative echocardiogram, (2) infective endocarditis, (3) rheumatic heart disease and (4) age < 18 years.

Pre-operative demographic and clinical data were retrospectively extracted from the hospital information system (HIX 6.3; ChipSoft BV, Amsterdam, The Netherlands) and the cardiology department’s electronic patient record (EPD-Vision^®^; Leiden University Medical Center, Leiden, The Netherlands). Collected variables included patient demographics, cardiovascular risk factors, clinical symptoms, comorbid cardiovascular conditions, concomitant surgical interventions, and follow-up information. All-cause mortality was ascertained through review of hospital records, which are linked to the national governmental death registry. Given the retrospective nature of this study and the use of anonymized clinical data, the Institutional Review Board waived the requirement for written informed consent.

### 2.2. Echocardiography

All patients underwent pre-operative transthoracic two-dimensional echocardiography using commercially available ultrasound systems (Vivid 5, Vivid 7, System 5, and E9; GE Healthcare, Vingmed, Horten, Norway). Image acquisition included standard parasternal and apical views, with patients examined in the left lateral decubitus position. Two-dimensional, M-mode, pulsed-wave and continuous-wave Doppler, as well as color Doppler imaging, were routinely performed. Echocardiographic data were digitally stored and subsequently analyzed offline using dedicated software (EchoPAC, versions 112, 202, and 203; GE Medical Systems, Horten, Norway).

The parasternal long-axis view was used to measure left ventricular (LV) dimensions, MV annular diameter, mitral annular disjunction (MAD) and the maximum left atrial (LA) diameter [10]. From the apical 2- and 4-chamber views, LV end-diastolic and end-systolic volumes were measured using the Simpson’s biplane method; LV ejection fraction was then calculated [10]. In addition to LV ejection fraction, LV global longitudinal strain (GLS) was measured as previously described [10] and presented in absolute values. The MR severity was graded according to current guidelines using a multi-parametric approach, including qualitative, semi-quantitative and quantitative parameters whenever available [11]. LA volumes were assessed at the end-systole from the apical 2- and 4-chamber views using the biplane Simpson method and were indexed to body surface area [10].

RV dimensions and areas, TV annular diameter, and maximal right atrial (RA) dimensions and volume were obtained from a focused apical 4-chamber view optimized for RV assessment [10,12]. RV fractional area change was calculated from end-diastolic and end-systolic RV areas [12]. RV pressure was determined using the peak velocity of the TR jet and the RA pressure based on the diameter and inspiratory collapse of the inferior vena cava to evaluate the systolic arterial pulmonary pressure [12]. To assess the RV systolic function, tricuspid annular plane systolic excursion (TAPSE) [13] was measured from M-mode in the focused RV 4-chamber apical view, and RV free wall strain was measured as previously described [10] and presented in absolute values. TR severity was graded according to current guidelines using a multi-parametric approach, including qualitative, semi-quantitative and quantitative parameters whenever available [11]. The (re)occurrence of significant TR (defined as TR grade ≥ 2) during follow-up was assessed using an echocardiogram performed approximately 1 year after surgery.

### 2.3. Definition of MV Prolapse

MV prolapse was defined as systolic displacement of one or both mitral leaflets of more than 2 mm beyond the mitral annular plane in the parasternal long-axis view [11]. Based on established criteria, MV prolapse was further classified into Barlow’s disease and fibroelastic deficiency (FED) based on the current literature. Barlow’s disease was characterized by bi-leaflet or multi-segment prolapse associated with excessive leaflet tissue, elongated or ruptured chordae, and significant annular dilatation [14,15]. FED was defined by single-segment prolapse, most commonly related to chordal rupture or flail, with thin leaflets and/or leaflet thickening confined to the prolapsing segment [14,15]. MAD was defined as a separation between the LA wall at the level of the MV junction and the LV free wall, with a cut-off value of >5 mm used to define significant disjunction [16].

### 2.4. Definition of TV Prolapse

The maximal systolic atrial displacement of the TV leaflets from the TV annular plane to the belly of the TV leaflets was measured in the focused RV 4-chamber apical view and the parasternal short-axis view at the level of the aortic valve. The focused RV 4-chamber apical view was available in all patients, but the quality of the parasternal short-axis view was, in the majority of the patients (94%), not optimal to assess TV leaflets. Therefore, the definition of TV prolapse was based on the focused RV 4-chamber apical view as leaflet displacement of >2 mm beyond the TV annulus (which showed previously to have good reproducibility [6]). In addition, the presence of tricuspid annular disjunction (TAD) was evaluated, defined as a separation between the RA wall at the level of the TV junction and the RV free wall of ≥1 mm [17].

### 2.5. Statistical Analysis

Categorical data are reported as counts and percentages. Continuous variables are presented as the mean ± standard deviation (SD) for normally distributed data, or as the median with the interquartile range (IQR) for non-normally distributed data. Normality of data distribution was evaluated using the Kolmogorov–Smirnov and Shapiro–Wilk tests. Depending on data type and distribution, comparisons between groups were performed using the chi-square test, unpaired Student’s t-test, Mann–Whitney U test, or Kruskal–Wallis test. Kaplan–Meier survival analysis was used to assess differences in all-cause mortality and the (re)occurrence of significant TR, with group comparisons performed using the log-rank test. Statistical analyses were conducted using SPSS software (version 29.0; IBM Corp., Armonk, NY, USA). A two-sided *p*-value < 0.05 was considered statistically significant.

## 3. Results

### 3.1. Clinical Characteristics

A total of 803 patients met the inclusion criteria, of which 87 (11%) were diagnosed at the pre-operative echocardiography with TV prolapse, while 716 patients (89%) showed no TV prolapse. Table 1 reports the clinical characteristics of the total study population and of the two groups, with and without TV prolapse. The mean age of the total population was 66 [57–73] years, and the majority of patients were male (63%). Patients with TV prolapse were more often diagnosed with Barlow’s disease compared to patients without TV prolapse (76% vs. 43%, *p* < 0.001). The two groups were similar in terms of age, symptoms, medication use, renal function, and comorbidities, except for the prevalence of coronary artery disease, which was more common in patients without TV prolapse who therefore underwent a concomitant coronary artery bypass graft (19% vs. 6%, *p* = 0.003) more often.

### 3.2. Echocardiographic Characteristics

Table 2 and Table 3 summarize the echocardiographic characteristics of the total population and of the patients with and without TV prolapse.

Considering the left side of the heart, patients with TV prolapse had larger LV dimensions, larger LV end-systolic volume and a more dilated LA compared to the patients without TV prolapse, despite the same severity of MR. When assessing LV function, LV ejection fraction was lower in patients with TV prolapse (63% [57–67] vs. 65% [60–69], *p* = 0.043), although still in the normal range, while LV GLS showed no significant difference between the two groups. Furthermore, patients with TV prolapse had a larger MV annulus (38 [36–42] mm vs. 33 [28–38] mm, *p* < 0.001) and more often MAD (31% vs. 19%, *p* = 0008).

Considering the right side of the heart, patients with TV prolapse had larger RV dimensions based on all three diameters (basal, mid, long) and both end-diastolic and end-systolic areas compared to the patients without TV prolapse. In addition, the TV annulus (41 [38–44] mm vs. 35 [31–39] mm, *p* < 0.001) and RA volume were also larger in patients with TV prolapse. The RV function, assessed with different measurements, was on average slightly better in patients with TV prolapse compared to patients without TV prolapse (TAPSE: 24 [22–27] mm vs. 23 [20–26] mm, *p* = 0.017 and RV strain: 27.2% [21.4–32.0] vs. 22.8% [18.0–27.3], *p* < 0.001). Importantly, patients with TV prolapse had moderate–severe and severe TR more often compared to the patients without TV prolapse (42% vs. 27%, *p* < 0.001). Based possibly on the above-mentioned characteristics, they also underwent TV annuloplasty (71% vs. 44%, *p* < 0.001) more often. However, when considering all patients with moderate—severe TR (n = 225), 37 (16% [107–164) [114–173] presented with TV prolapse, the other 188 patients (84%) showed no TV prolapse and had therefore secondary TR.

### 3.3. TV Prolapse and TAD

**Figure 1:**
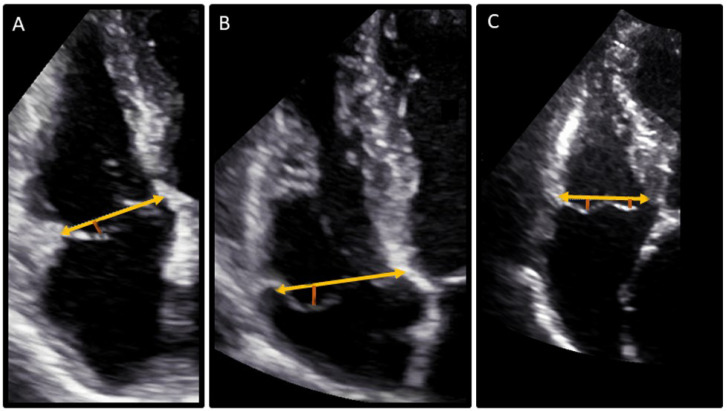
Examples of patients with TV prolapse assessed from an apical RV-focused view. Panel (**A**–**C**) show patients with a mural leaflet prolapse, as depicted with the red line; the orange line shows the tricuspid annulus from which the measurement starts. Panel (**C**) shows a patient with a mural and septal leaflet prolapse, as depicted with the two red lines coming from the tricuspid valve annulus (orange line).

Of the 87 patients with MV and TV prolapse, 63 patients had a prolapse of the mural leaflet only, eight patients had a prolapse of the septal leaflet only and 16 patients had a prolapse of the mural and septal leaflet (Figure 1). The median atrial displacement of the mural leaflet was 3.0 mm [IQR: 2.8–3.7 mm], and the median atrial displacement of the septal leaflet was 3.0 mm [IQR: 2.7–3.5 mm]. In addition, the presence of TAD was assessed in the whole population and observed only in 11 patients (1%), all with TV prolapse.

### 3.4. Surgical TV Annuloplasty

Out of 803 patients, 379 (47%) underwent concomitant TV annuloplasty, mainly according to the criteria of the ESC guidelines [18], which included at least moderate TR or significant TV annulus dilation together with RV dilatation and dysfunction. The surgical approach was tailored to the individual patient and included, in almost all patients, ring annuloplasty. Of these 379 patients, 62 (16%) had TV prolapse, and these patients were implanted with a slightly larger ring size (33 ± 2 vs. 32 ± 2, *p* = 0.003) compared to the patients without TV prolapse.

At a follow-up echocardiography (median time 13 months), of the 87 patients with TV prolapse (median follow-up time 52 months), only six (7%) of them presented a significant TR (TR grade ≥ 2), of which three had received TV annuloplasty (therefore 5% recurrence), and three did not undergo TV annuloplasty (therefore 12% of recurrent TR) (*p* = 0.233). Similarly, among the 716 patients without TV prolapse (median follow-up time 12 months), 87 (12%) presented with significant TR during follow-up, of which 14 received TV annuloplasty (4.5% TR recurrence), and 73 did not undergo TV annuloplasty (18% of recurrent TR) (*p* < 0.001).

Of note, during follow-up, the prevalence of recurrent MR was very low; only 20 patients (2.5%) developed grade 3 or 4 MR, and with no significant difference among patients with (n = 5/87, 6%) and without TV prolapse (n = 15/716, 2%) (*p* = 0.057).

### 3.5. Outcome

**Figure 2:**
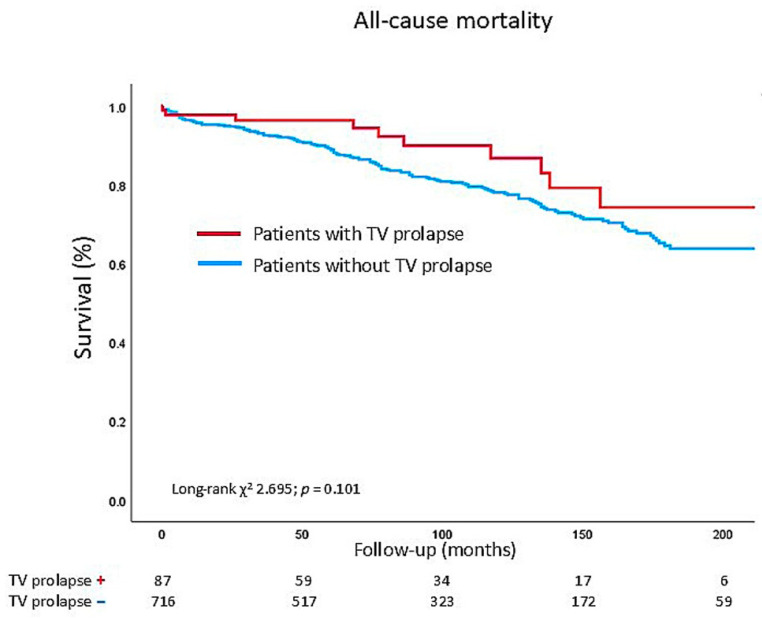
Survival analysis according to MV prolapse patients with and without TV prolapse. Kaplan–Meier curve estimated for cumulative event rates of all-cause mortality. TV = tricuspid valve.

During a median clinical follow-up time of 89 months [IQR: 44–145 months], 164 patients died, and all-cause mortality was not significantly different between patients with or without TV prolapse after surgery (log-rank chi-square 2.695; *p* = 0.101) (Figure 2). Of those 164 patients, 10 patients had a TV prolapse, and 154 patients had no TV prolapse. Of note, comparing the all-cause mortality rate between TV prolapse patients with and without concomitant TV annuloplasty also revealed no significant difference (log-rank chi-square 2.676; *p* = 0.102).

## 4. Discussion

The main findings of the current study can be summarized as follows: (1) in a large cohort of patients with significant primary MR undergoing surgery, 11% of the patients also presented echocardiographic criteria for TV prolapse, which was more frequent in patients diagnosed with Barlow’s disease, (2) TV prolapse patients had also more frequently significant TR, a larger right chamber size and TV annulus (with TAD) and therefore underwent TV annuloplasty more often, and (3) no difference in outcomes after surgery, in terms of TR progression or all-cause mortality, were observed between patients with and without TV prolapse.

### 4.1. Prevalence of Tricuspid Valve Prolapse

The prevalence of TV prolapse can significantly differ according to the patient population, but also to the definition and the imaging modality used for diagnosis. Lorinksy et al. [6] proposed to define TV prolapse with a cut-off value of >2 mm in the apical 4-chamber and parasternal short-axis view and >4 mm in the right ventricular inflow view. In their study, TV prolapse was identified in 0.4% of a general cohort of patients referred for transthoracic echocardiography in their medical center; however, 75% of the patients with TV prolapse also had MV prolapse. Using the same definition, Donia et al. [19] reported a prevalence of TV prolapse of 41% in a small cohort of patients with MV prolapse but without significant MR. More recently, Margonato et al. [2] evaluated (using both transthoracic and transesophageal echocardiography) the prevalence of different TR etiologies in a large cohort of patients with severe degenerative MR and diagnosed TV prolapse in 55% of them. Although the authors suggested that TV prolapse is the most frequent etiology of TR in these patients, only 30% of them had more than mild TR. In the current study a lower percentage of TV prolapse was observed, possibly in relation to the higher percentage of FED patients and to the use of only transthoracic echocardiography. Also, in our cohort, the etiology of TR was in the large majority of patients secondary to increased pulmonary pressures or RA and RV remodeling, which is in agreement with other previous studies where primary TR is relatively rare in patients with MV prolapse [3]. However, the presence of TV prolapse was associated with a higher prevalence of significant TR, suggesting an important role of the TV leaflet abnormalities in the development of TR. In a similar study using magnetic resonance imaging in patients with MV prolapse, TV prolapse was observed in approximately 35% of patients, 24% of whom showed significant TR [7].

### 4.2. TV Prolapse and Associated Echocardiographic Characteristics

In our cohort, patients with TV prolapse were more often diagnosed with Barlow’s disease (more often bi-leaflet prolapse, larger MV annulus diameter and more often MAD), which is consistent with previous studies. In the study of Guta et al. [7], TV prolapse was more often present in patients with bi-leaflet MV prolapse compared to single-leaflet MV prolapse. Moreover, in the study of Margonato et al. [2], TV prolapse was also seen more often in patients with Barlow’s disease (myxomatous degeneration and bi-leaflet prolapse). On the other hand, in the study of Donia et al. [19], MV prolapse (but without significant MR) patients with and without TV prolapse showed no differences in the occurrence of single or bi-leaflet MV prolapse, occurrence of MAD or systolic curling. Taking these studies together, a possible association between MV prolapse severity and the occurrence of TV prolapse could be related to the degenerative (myxomatous) abnormalities, which might be genetically determined or developmental [20]. In this regard, the presence of TAD might also suggest a similar etiology of TV and MV prolapse. In a recent study by Aabel et al. [17], 84 patients with MAD on cardiac magnetic resonance imaging were evaluated for the presence of TAD, which was found in 42 (50%) of them. This prevalence was higher than the prevalence of TAD in our study. However, when echocardiography images were analyzed in the study of Aabel et al. [17], TAD was seen only in 2 of the 42 patients diagnosed with TAD on cardiac magnetic resonance imaging.

In our cohort, patients with TV prolapse also showed larger LV and LA volumes (despite similar MR severity), and larger RV, RA and TV annulus size compared to patients without TV prolapse. These findings might be explained by the fact that patients with TV prolapse were more often diagnosed with grade 2 and 3 TR compared to the patients without TV prolapse, who more often had grade 1 TR. However, a disproportionate remodeling of the cardiac chambers (namely atria and annulus) to the severity of the atrio-ventricular regurgitation has also been described in Barlow’s patients, which were the most frequent in cases of TV prolapse [3,21]. Interestingly, RV function appeared to be slightly better in patients with TV prolapse compared to patients without TV prolapse, a finding that has also been reported previously by Donia et al [19]. However, parameters such as TAPSE and RV strain are still load-dependent and may therefore be influenced by increased preload in the presence of more significant TR. In addition, intrinsic connective tissue abnormalities of the TV apparatus (hypermobile) may also contribute to these findings. These results should therefore be interpreted with caution.

### 4.3. Surgical Intervention and Outcome

In this study, 47% of the patients had concomitant TV annuloplasty, indicated mostly based on the current guidelines’ recommendations, and this percentage was higher in the patients who also had TV prolapse (71%) compared to the patients without TV prolapse (44%). This difference could be explained by more severe TV annulus dilatation and TR severity, both of which are criteria for combined TV surgery. During follow-up, no difference was observed between patients with and without TV prolapse in terms of recurrence or residual TR, although this is possibly related to the fact that both groups were similarly treated with TV annuloplasty. The identification of TV prolapse is still of relevance with potential implications in the surgical technique (as annuloplasty might not always be sufficient or the ring size could be adapted) and also a better understanding of patients’ disease. Similarly, in terms of all-cause mortality, no significant differences were observed related to the presence of TV prolapse. Possibly patients with Barlow’s disease (therefore with both MV and TV prolapse) were also more closely monitored anyway regarding risk for arrhythmias or endocarditis.

### 4.4. Study Limitations

Several study limitations should be mentioned. First, the current study was retrospective with limitations inherent to its design; prospective, larger studies with longer (echocardiographic) follow-up are advocated to confirm these findings. Second, our findings are specific to a cohort of patients with MV prolapse and significant MR referred for MV intervention in our tertiary center. Third, transthoracic echocardiography was chosen for the TV prolapse diagnosis as the first-line and most widely available imaging technique; however, the parasternal short-axis view was not used in the majority of the patients due to the suboptimal quality of this view, therefore, it could be possible that TV prolapse was missed in some patients resulting in an underestimation of the prevalence of TV prolapse in our cohort. Also, magnetic resonance to evaluate the presence of MAD and TAD and better assess MV and TV prolapse was not systematically performed.

## 5. Conclusions

In our large study cohort with patients with MV prolapse and significant MR, we described the prevalence of TV prolapse and its association with a more severe phenotype in terms of cardiac remodeling and TR severity. However, when successfully treated, TV prolapse is not associated with worse outcomes after surgery, also in terms of TR progression.

## Figures and Tables

**Table 1:** Clinical characteristics of the total population and among patients with and without TV prolapse.

	Total N = 803	TV Prolapse N = 87	No TV Prolapse N = 716	*p*-Value
Age at surgery, years	66 [57–73]	65 [54–73]	66 [57–74]	0.245
Male, n (%)	531 (66)	55 (63)	476 (67)	0.544
Etiology, n (%)				<0.001
Fibro-elastic deficiency	432 (54)	21 (24)	411 (57)	
Barlow’s disease	371 (46)	66 (76)	305 (43)	
Palpitations, n (%)	283 (35)	29 (33)	254 (36)	0.693
NYHA ≥ 3, n (%)	194 (24)	19 (22)	175 (24)	0.583
Hypertension, n (%)	327 (41)	28 (32)	299 (42)	0.086
Diabetes, n (%)	24 (3)	4 (5)	20 (3)	0.351
History of smoking, n (%)	280 (35)	27 (31)	253 (35)	0.516
Coronary artery disease, n (%)	175 (22)	7 (8)	168 (24)	0.001
Chronic obstructive pulmonary disease, n (%)	53 (7)	5 (6)	48 (7)	0.718
Atrial fibrillation, n (%)	288 (36)	34 (39)	254 (35)	0.773
Ventricular arrhythmias, n (%)	117 (15)	15 (17)	102 (14)	0.455
PM/ICD, n (%)	25 (3)	2 (2)	23 (3)	0.639
eGFR, mL/min/1.73 m^2^	73 ± 18	75 ± 16	72 ± 19	0.273
Medication, n (%)				
Oral anticoagulants	181 (23)	25 (29)	156 (22)	0.301
Betablocker	238 (30)	26 (30)	212 (30)	0.606
AT2/ACE-inhibitor	289 (36)	27 (31)	262 (37)	0.074
Calcium antagonist	59 (7)	9 (10)	50 (7)	0.379
Diuretics	233 (29)	28 (32)	205 (29)	0.873
Concomitant surgery, n (%)				
TV annuloplasty	379 (47)	62 (71)	317 (44)	<0.001
CABG	139 (17)	5 (6)	134 (19)	0.003

ACE = angiotensin converting enzyme, AT2 = angiotensin II, CABG = coronary artery bypass graft, ICD = implantable cardioverter defibrillator, eGFR = estimated glomerular filtration rate, NYHA = New York Heart Association, PM = pacemaker, and TV = tricuspid valve.

**Table 2:** Baseline echocardiographic left heart parameters of the total population and among patients with and without TV prolapse.

	Total N = 803	TV Prolapse N = 87	No TV Prolapse N = 716	*p*-Value
LV end-diastolic diameter, mm	55 ± 7	58 ± 7	54 ± 7	<0.001
LV end-systolic diameter, mm	34 ± 7	35 ± 7	33 ± 7	0.025
LV end-diastolic volume, mL	134 [109–165]	139 [114–173]	133 [107–164]	0.110
LV end-systolic volume, mL	47 [36–61]	51 [41–68]	47 [35–61]	0.012
LV ejection fraction, %	65 [60–69]	63 [57–67]	65 [60–69]	0.043
LV GLS, %	21.1 ± 4.1	21.5 ± 4.1	21.0 ± 4.1	0.296
LA end-systolic diameter, mm	45 [40–49]	45 [40–53]	45 [40–49]	0.275
LA volume index, mL/m^2^	51 [40–64]	58 [45–80]	51 [39–63]	0.001
MV annulus diameter, mm	34 [29–39]	38 [36–42]	33 [28–38]	<0.001
Mitral annular disjunction *, n (%)	162 (20)	27 (31)	135 (19)	0.008
MR grade, n (%)				0.897
moderate—severe	243 (30)	25 (29)	218 (30)	
severe	560 (70)	62 (71)	498 (70)	

GLS = global longitudinal strain, LA = left atrial, LV = left ventricular, MR = mitral regurgitation, and MV = mitral valve. * Cut-off value ≥ 5 mm.

**Table 3:** Baseline echocardiographic right heart parameters of the total population and among patients with and without TV prolapse.

	Total N = 803	TV Prolapse N = 87	No TV Prolapse N = 716	*p*-Value
RV basal diameter, mm	39 [35–44]	44 [38–47]	39 [35–43]	<0.001
RV mid diameter, mm	21 [18–25]	22 [19–27]	21 [18–25]	0.033
RV long diameter, mm	70 [63–77]	73 [66–80]	70 [63–76]	0.005
RV end-diastolic area, cm^2^	19 [16–23]	21 [17–24]	19 [16–23]	0.002
RV end-systolic area, cm^2^	11 [9–14]	12 [10–14]	11 [9–14]	0.006
RV fractional area change, %	41 [36–47]	40 [36–46]	41 [36–47]	0.644
RV free wall strain, %	23 [−28–18]	27.2 [−32–21.4]	22.8 [−27.3–18.0]	<0.001
TAPSE, mm	23 [20–26]	24 [22–27]	23 [20–26]	0.017
TV annulus diameter, mm	35 [31–39]	41 [38–44]	35 [31–39]	<0.001
Tricuspid annular disjunction *, n (%)	11 (1)	11 (12)	0 (0)	<0.001
Systolic pulmonary pressure, mmHg	33 [26–45]	31 [26–42]	33 [26–45]	0.213
RA maximum diameter, mm	50 [46–57]	51 [47–56]	50 [45–57]	0.462
RA end-systolic volume, mL	43 [31–62]	55 [39–78]	42 [31–59]	<0.001
TR grade, n (%)				0.016
mild	578 (72)	50 (58)	528 (74)	
moderate	175 (22)	27 (31)	148 (21)	
moderate—severe	43 (5)	9 (10)	34 (5)	
severe	7 (1)	1 (1)	6 (1)	

RA = right atrial, RV = right ventricular, TAPSE = tricuspid annular plane systolic excursion, TR = tricuspid regurgitation, and TV = tricuspid valve. * Cut-off value ≥ 1 mm.

## Data Availability

Data available upon request due to restrictions (privacy).

## Abbreviations

The following abbreviations are used in this manuscript:

FED	fibroelastic deficiency
GLS	global longitudinal strain
LA	left atrial
LV	left ventricular
MAD	mitral annular disjunction
MR	mitral regurgitation
MV	mitral valve
RA	right atrial
RV	right ventricular
TAD	tricuspid annular disjunction
TAPSE	tricuspid annular plane systolic excursion
TR	tricuspid regurgitation
TV	tricuspid valve
